# Robust Tightly Coupled Pose Measurement Based on Multi-Sensor Fusion in Mobile Robot System

**DOI:** 10.3390/s21165522

**Published:** 2021-08-17

**Authors:** Gang Peng, Zezao Lu, Jiaxi Peng, Dingxin He, Xinde Li, Bin Hu

**Affiliations:** 1 Key Laboratory of Image Processing and Intelligent Control, Ministry of Education, Wuhan 430070, China; penggang@hust.edu.cn (G.P.); M201672539@hust.edu.cn (Z.L.); hedingxin@mail.hust.edu.cn (D.H.); xindeli@seu.edu.cn (X.L.); 2 School of Artificial Intelligence and Automation, Huazhong University of Science and Technology, Wuhan 430070, China; 3 School of Automation, Southeast University, Nanjing 210096, China; 4 Shantui Construction Machinery Co., Ltd., Jining 272000, China; binhuyan@163.com

**Keywords:** multi-sensor fusion, robot pose measurement, simultaneous localization and mapping, visual inertia system

## Abstract

Currently, simultaneous localization and mapping (SLAM) is one of the main research topics in the robotics field. Visual-inertia SLAM, which consists of a camera and an inertial measurement unit (IMU), can significantly improve robustness and enable scale weak-visibility, whereas monocular visual SLAM is scale-invisible. For ground mobile robots, the introduction of a wheel speed sensor can solve the scale weak-visibility problem and improve robustness under abnormal conditions. In this paper, a multi-sensor fusion SLAM algorithm using monocular vision, inertia, and wheel speed measurements is proposed. The sensor measurements are combined in a tightly coupled manner, and a nonlinear optimization method is used to maximize the posterior probability to solve the optimal state estimation. Loop detection and back-end optimization are added to help reduce or even eliminate the cumulative error of the estimated poses, thus ensuring global consistency of the trajectory and map. The outstanding contribution of this paper is that the wheel odometer pre-integration algorithm, which combines the chassis speed and IMU angular speed, can avoid the repeated integration caused by linearization point changes during iterative optimization; state initialization based on the wheel odometer and IMU enables a quick and reliable calculation of the initial state values required by the state estimator in both stationary and moving states. Comparative experiments were conducted in room-scale scenes, building scale scenes, and visual loss scenarios. The results showed that the proposed algorithm is highly accurate—2.2 m of cumulative error after moving 812 m (0.28%, loopback optimization disabled)—robust, and has an effective localization capability even in the event of sensor loss, including visual loss. The accuracy and robustness of the proposed method are superior to those of monocular visual inertia SLAM and traditional wheel odometers.

## 1. Introduction

At present, the influential simultaneous localization and mapping (SLAM) systems with monocular vision include ORB-SLAM2 [1], LSD-SLAM [2], DSO [3], and SVO [4]. Among them, ORB-SLAM2 [4] has high adaptability and strong robustness to indoor and outdoor environments. However, the algorithm itself is based on pure vision. When there are no visual features, the accuracy and robustness of the pose estimation will decrease rapidly, and the algorithm may fail. Therefore, in the follow-up development of visual SLAM, in order to overcome the shortcomings of pure vision, the strategy of multisensor fusion is adopted.

Accordingly, sensors with scale measurement capabilities and monocular vision sensors are used to perform fusion vision SLAM to increase accuracy and robustness. Relatively stable and reliable solutions can be obtained with lasers [5]; however, at present, this method is generally only suitable for large-scale scenarios, such as unmanned driving, and is unsuitable for applications with limited costs. The inertial measurement unit (IMU) has become a generally accepted option. However, it exhibits a non-negligible cumulative error if run for a long period of time [6], especially in visually restricted conditions without texture or under weak illumination, in which case the visual mile cannot be used to correct the IMU error. In [7], the scale observability of a monocular visual inertial odometer on a ground mobile robot was analyzed in detail. When the robot moved at a constant speed, due to the lack of acceleration excitation, the constraint on the scale was lost, resulting in a gradual increase in the scale uncertainty and positioning error.

A wheel speed inertial odometer was integrated with a monocular visual odometer using the extended Kalman filter (EKF) [8,9], assuming the robot to be running on an ideal plane, and 3-DOF pose measurement was performed. The wheel speed inertial odometer uses wheel speed measurement for EKF status prediction; the angular speed measurement is integrated for dead reckoning; and the visual odometer method is used for an EKF measurement update. In the above-mentioned EKF-based loose coupling method, when the visual odometer cannot accurately calculate the pose due to insufficient visual characteristics, some proportion of its output in the filter will fall, resulting in ineffective visual observation. Consequently, the accuracy is reduced. VINS-Mono used tightly coupled nonlinear optimization [10,11]. By combining the IMU pre-integral measurement and visual measurement in a tightly coupled form to achieve the maximum posterior probability, we use the nonlinear optimization method to reach the optimal state. An open-source visual inertia SLAM algorithm VINS-Fusion [12,13] was developed based on VINS-Mono, supporting multiple sensor combinations (binocular camera + IMU; monocular camera + IMU; binocular camera only); GPS is used to improve the accuracy of the global path. In pursuit of better performance, wheel speed is used in SLAM system [14]—the wheel speed sensor and the visual odometer were integrated tightly. In this way, they make least-squares rather than optimization. However, they do not consider the unreliability of wheel speed. When a robot moves on uneven surfaces, or a wheel slip occurs, an incorrect wheel speed measurement will significantly affect the scale accuracy, and can potentially lead to system failure. In [15], a batch optimization framework with formulated edges was proposed. Moreover, tightly coupled nonlinear optimization methods were used to integrate vision, IMU, and wheel speed, and to perform pose. The error cost function was composed of vision errors, inertial measurement errors, and wheel speed sensor dead reckoning errors. In addition, assuming that the vehicle was moving on an approximate plane, a “soft” plane constraint term was added to the error cost function. While the robot is running with fixed acceleration or going straight, we do not know how long it moves in real time or what the direction is. Therefore, the encoder and soft plane constraints will make the task easier.

According to whether the visual feature points are added to the state vector of the filter or the optimization equation, the visual inertial odometry method based on filtering can be divided into two types: loose coupling and tight coupling. Further, the loose coupling method is divided into two parts: the visual odometer and state estimator. The visual odometer tracks the feature points and calculates the current camera pose, and the state estimator uses the IMU to measure and adjust the camera pose obtained by the visual odometer. As the visual features are invisible to the state estimator, they cannot be adjusted based on the IMU measurement, and the poor position accuracy of the visual features causes the accuracy of the visual odometer to decline. As a result, the correlation between measurements is not fully utilized for pose measurement, thereby reducing the accuracy. In the tight coupling method, the carrier pose and position of the feature point are used as variables to be estimated, and the filter method or optimized method is used for estimation.

Thus far, for the existing visual inertia SLAM algorithm, when the robot is moving at a constant speed, or purely rotating, and encounters scenes with insufficient visual features, the problems of low accuracy and poor robustness arise. Tightly coupled wheel speed information can help solve this problem. However, there are few studies on multi-sensor fusion SLAM for wheeled mobile robots based on vision, inertia, and wheel speed measurements that are tightly coupled and optimized. There is no complete solution similar to ORB-SLAM. Therefore, research on tightly coupled monocular visual odometer combined with wheel speed measurement is significant. Therefore, we use IMU and wheel odometry to make a monocular visual odometry system to gain the scale reconstruction.

The main novelty and contributions of this paper include:

1. A multi-sensor fusion SLAM algorithm using monocular vision, inertial measurement, and wheel speed measurement is proposed. The wheel speed and IMU angular velocity pre-integration of the wheel odometer can avoid the repeated integration caused by the linearization point change in the iterative optimization process.
2. Based on the state initialization of the wheel odometer and IMU, the initial state value required by the state estimator can be quickly and reliably calculated in both stationary and moving states.

The method in this paper solves the problem of poor pose estimation accuracy caused by the weak observability of monocular visual inertial SLAM, and further improves the robustness of pose estimation.

## 2. Multi-Sensor State Estimation Based on Tight Coupling Optimization

### 2.1. SLAM Based on Multi-Sensor Fusion

**Figure 1:**
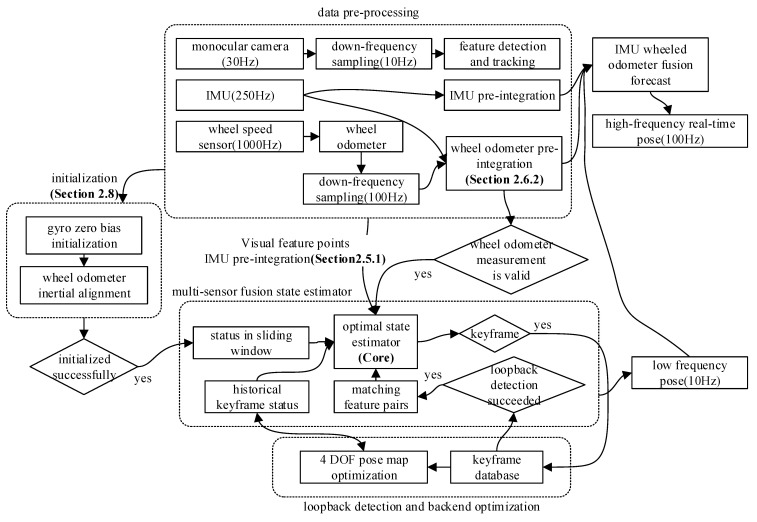
Block diagram of monocular vision inertial SLAM.

The multi-sensor fusion state estimator in this study uses monocular vision, IMU, and wheel odometer measurements based on feature point optical flow tracking [16]. None of these sensors can measure the absolute pose. Therefore, the multi-sensor fusion state estimator, as an odometer algorithm, has an unavoidable cumulative error. Therefore, we used key frame selection, loop detection [17], and back-end optimization algorithms based on the VINS-Mono framework and applied them to the multi-sensor fusion state estimator to form a complete SLAM system. Figure 1 shows a system block diagram of the SLAM method.

In a multi-sensor state estimation process, the main data processing and analysis processes include raw sensor input, data pre-processing (including calibration compensation, time alignment, and pre-fused wheel odometer), state initialization, state estimation problem solving, loopback detection, and backend optimization. The processes of optimal state estimator, wheel odometer pre-integration, and initialization reflect the contributions and innovations of this paper. The rest is our flexible use of existing methods. Section 2.2, Section 2.3, Section 2.4, Section 2.5, Section 2.6 and Section 2.7 analyze the core multi-sensor fusion state estimation problem and the pre-integration process of IMU and wheel speed. Section 2.8 analyzes the initialization process of the state.

### 2.2. Variables to Be Estimated and Observation

Considering that the IMU zero offset always exists, when defining the variable X to be estimated, the IMU zero offset of each key frame needs to be included. Therefore, in the k key frame, we have:

(1) $$\begin{array}{c} X={\left\{x_{k},\lambda_{l}\right\}}_{k\in K,l\in ℒ}\\ x_{k}=\left[p_{B_{k}}^{W},v_{B_{k}}^{W},q_{B_{k}}^{W},b_{ak},b_{gk}\right] \end{array}$$

For the IMU, $x_{k}\in R^{16\times 1}$ is the state, $p_{B}^{W}\in R^{3\times 1}$ denotes the position, $q_{B}^{W}\in R^{4\times 1}$ is the attitude (quaternion form), $v_{B}^{W}\in R^{3\times 1}$ denotes the speed, and $b_{a}\in R^{3\times 1}$ and $b_{g}\in R^{3\times 1}$ are the accelerometer and gyroscope bias, respectively. K is the key frames and ℒ is the feature points. Further, $\lambda_{l}$ is the inverse depth of ℒ (the inverse of the Z-axis coordinate).

Additionally, pre-fusion wheel odometer observations are added based on VINS-Mono. The original wheel odometer is defined as:

(2) $${\hat{m}}_{odomk}≜{\left[\begin{array}{ccc} p_{xk} & p_{yk} & \theta_{k} \end{array}\right]}^{T}$$

where {$p_{xk}$ $p_{yk}$} is the coordinate of the wheel odometer relative to the starting point and $\theta_{k}$ is the angle of rotation relative to the starting direction. Therefore, the observation Z used to constrain the X:

(3) $$Z={\left\{Z_{C_{i}},{ℬ}_{ij},O_{ij}\right\}}_{\left(i,j\right)\in K}$$

where visual feature point observation $Z_{C_{i}}={\left\{{\hat{z}}_{il}\right\}}_{l\in {ℒ}_{i}}$, containing all feature points ${ℒ}_{i}$ observed under the i key frame; ${ℬ}_{ij}={\left\{{\hat{a}}_{t},{\hat{\omega }}_{t}\right\}}_{t_{i}\leq t\leq t_{j}}$ is IMU pre-integration of accelerated speed and angular speed; and $O_{ij}={\left\{\Delta {\hat{m}}_{odomt},{\hat{a}}_{avgt},{\hat{\omega }}_{avgt}\right\}}_{t_{i}\leq t\leq t_{j}}$ is pre-fusion wheel odometer.

### 2.3. Optimal Estimation Problem

The maximum posterior estimation and Bayes’ theorem shows:

(4) $$X^{*}=\underset{X}{\mathrm{argmax}}p\left(Z|X\right)p\left(X\right)$$

Here, p(Z|X) is the conditional probability of observing the occurrence of Z under the existed state X, which can be obtained by the observation equation and the covariance. p(X) is the prior probability (edge probability) of X. According to formula (3), we have:

(5) $$\begin{array}{cc} p\left(Z|X\right)p\left(X\right) & =\;p\left(X\right)\underset{\left(i,j\right)\in K}{\prod }p(Z_{C_{i}},{ℬ}_{ij},O_{ij}\;|\;X)\\ & =\;p\left(X\right)\underset{i\in K}{\prod }\underset{l\in ℒ}{\prod }p\left({\hat{z}}_{il}|x_{i},\lambda_{l}\right)\underset{\left(i,j\right)\in K}{\prod }p({\left\{{\hat{a}}_{t},{\hat{\omega }}_{t}\right\}}_{t_{i}\leq t\leq t_{j}}|x_{i},x_{j})\\ & \times \;\underset{\left(i,j\right)\in K}{\prod }p({\left\{\Delta {\hat{m}}_{odomt},{\hat{a}}_{avgt},{\hat{\omega }}_{avgt}\right\}}_{t_{i}\leq t\leq t_{j}}|x_{i},x_{j}) \end{array}$$

We use the least-squares method to find the maximum posterior estimate, and use Mahalanobis distance to measure the difference between the residual and the covariance matrix.

(6) $$\begin{array}{ll} X^{*} & =\;\underset{X}{\mathrm{argmin}}\{\|r_{p}-H_{p}X{\|}^{2}+\underset{i\in K}{\sum }\underset{l\in ℒ}{\sum }\rho \left(\|r_{C}{\left({\hat{z}}_{il},x_{i},\lambda_{l})\right\|}_{\Sigma_{C_{il}}}^{2}\right)\\ & +\;\underset{\left(i,j\right)\in K}{\sum }\|r_{ℬ}{\left({\left\{{\hat{a}}_{t},{\hat{\omega }}_{t}\right\}}_{t_{i}\leq t\leq t_{j}},x_{i},x_{j})\right\|}_{\Sigma_{{ℬ}_{ij}}}^{2}\\ & +\;\underset{\left(i,j\right)\in K}{\sum }\rho \left(\|r_{O}{\left({\left\{\Delta {\hat{m}}_{odomt},{\hat{a}}_{avgt},{\hat{\omega }}_{avgt}\right\}}_{t_{i}\leq t\leq t_{j}}|x_{i},x_{j})\right\|}_{\Sigma_{O_{ij}}}^{2}\right)\} \end{array}$$

Here, $r{\left\|\left(\cdot \right)\right\|}_{\Sigma }^{2}$ is the Mahalanobis distance and Σ is the covariance matrix. $\left\{r_{p},H_{p}\right\}$ is the prior information from marginalization; $r_{C}\in R$ is the visual residual; $r_{O}\in R^{3\times 1}$ is the wheel odometer residual; and $r_{ℬ}\in R^{15\times 1}$ is the IMU residual. In addition, the Huber loss function ρ [18] is used to improve the robustness, where ρ is

(7) $$\rho \left(s\right)=\{\begin{array}{c} 1\;s\geq 1\\ 2\sqrt{s}-1\;s<1 \end{array}$$

### 2.4. Visual Measurement Constraints

The specific process is as follows: while the feature point l is first observed in the key frame i, it is recorded and tracked. Its spatial pose is defined as a function of the key frame i pose $\left(p_{C_{i}},q_{C_{i}}\right)$ and the inverse depth $\lambda_{l}$ of the feature point. While the feature point l is observed again in the key frame j, a visual residual term is generated. The residual term $r_{C_{jl}}$ represents the error of the feature point *l* in the position of j. It is also called the re-projection error, which is a function of the key frame i pose $\left(p_{C_{i}},q_{C_{i}}\right)$.

(8) $$r_{C}\left({\hat{z}}_{jl},X\right)=r_{C_{jl}}\left(p_{B_{i}}^{W},p_{B_{j}}^{W},q_{B_{i}}^{W},q_{B_{j}}^{W},\lambda_{l}\right)={\left[\begin{array}{cc} b_{1} & b_{2} \end{array}\right]}^{T}\cdot \left({{\hat{P}}_{l}}^{C_{j}}-\frac{{P_{l}}^{C_{j}}}{\left\|{P_{l}}^{C_{j}}\right\|}\right)$$

Here,

(9) $${{\hat{P}}_{l}}^{C_{j}}=\pi_{c}^{-1}\left({\hat{z}}_{il}\right)=\pi_{c}^{-1}\left(\left[\begin{array}{c} {\hat{u}}_{l}^{c_{j}}\\ {\hat{v}}_{l}^{c_{j}} \end{array}\right]\right)\;{P_{l}}^{C_{j}}=(R_{B}^{C}\left(R_{W}^{B_{j}}\left(R_{B_{i}}^{W}\left(R_{C}^{B}\cdot \frac{1}{\lambda_{l}}\cdot \pi_{c}^{-1}\left(\left[\begin{array}{c} u_{l}^{c_{u}}\\ v_{l}^{c_{u}} \end{array}\right]\right)+p_{C}^{B}\right)+p_{B_{i}}^{W}-p_{B_{j}}^{W}\right)-p_{C}^{B}\right)$$

In the formula, $R_{{ℱ}_{2}}^{{ℱ}_{1}}\in SO\left(3\right)$ represents the rotation matrix from ${ℱ}_{2}$ coordinate system to ${ℱ}_{1}$ coordinate system; ${{\hat{P}}_{l}}^{c_{j}}\in R^{2\times 1}$ is the position where the feature point *l* is projected onto the unit ball in the key frame *j*; $\pi_{c}^{-1}$ is the back projection function, which can project the pixel coordinates into the camera coordinate system $C_{j};\;{P_{l}}^{C_{j}}\in R^{2\times 1}$ is the position projected on the unit ball in the key frame j; to compare the error with ${P_{l}}^{C_{j}}$, it needs to be transformed into the camera coordinate system *C_j_* of the key frame *j*; and $\left[\begin{array}{cc} b_{1} & b_{2} \end{array}\right]$ are the two orthogonal base vectors on the tangent plane of the unit ball and the projection lines with the feature points in the orthogonal direction.

### 2.5. IMU Constraints

In the visual odometer method based on bundle adjustment, the state of the carrier is optimized and visual measurement is used to constrain. The IMU measurement between frames is added as a constraint on the optimization framework, thereby improving robustness.

#### 2.5.1. IMU Pre-Integration

To reduce the complicated operation caused by reintegration, we used the IMU pre-integration method [19] to fuse IMU measurements between two consecutive key frames.

#### 2.5.2. Residual Term

The IMU pre-integration processes the IMU measurement for a continuous period based on the given IMU zero offset and obtains the relative pose constraint between the initial and end states of the time period. The IMU pre-integration residual term is defined as:

(10) $$\begin{array}{cc} & r_{ℬ}\left({\left\{{\hat{a}}_{t},{\hat{\omega }}_{t}\right\}}_{t_{k}\leq t\leq t_{k+1}},x_{k},x_{k+1}\right)\\ & =\left[\begin{array}{c} \delta \alpha_{B_{k}+1}^{B_{k}}\\ \delta \beta_{B_{k}+1}^{B_{k}}\\ \begin{array}{c} \delta \theta_{B_{k}+1}^{B_{k}}\\ \delta b_{a}{}_{B_{k}+1}^{B_{k}}\\ \delta b_{g}{}_{B_{k}+1}^{B_{k}} \end{array} \end{array}\right]=\left[\begin{array}{c} R{\left\{q_{B_{k}}^{W}\right\}}^{T}\left(p_{B_{k}+1}^{W}-p_{B_{k}}^{W}-v_{B_{k}}^{W}\Delta t+\frac{1}{2}g\Delta t^{2}\right)-{\hat{\alpha }}_{B_{k}+1}^{B_{k}}\\ R{\left\{q_{B_{k}}^{W}\right\}}^{T}\left(v_{B_{k}+1}^{W}-v_{B_{k}}^{W}+g\Delta t\right)-{\hat{\beta }}_{B_{k}+1}^{B_{k}}\\ \begin{array}{c} 2{\left\{{\hat{q}}_{B_{k}+1}^{B_{k}}*⊗q_{B_{k}}^{W}*⊗q_{B_{k}+1}^{W}\right\}}_{xyz}\\ b_{aB_{k+1}}-b_{aB_{k}}\\ b_{gB_{k+1}}-b_{gB_{k}} \end{array} \end{array}\right] \end{array}$$

The random distribution of the residual term $r_{ℬ}$ conforms to $N\left(0,\Sigma_{B}\right)$ and $\Sigma_{B}$ is obtained by updating the covariance equation. $\delta \theta_{B_{k}+1}^{B_{k}}\in R^{3\times 1}$ is three-dimensional small perturbation and δ represents the error term. The IMU pre-integration provides constraints on the variables to be optimized contained in the two key frames before and after. In the process of nonlinear optimization, the essence of the constraint is to provide the direction and gradient of the variable to be optimized by calculating the Jacobian matrix of the residual. As the direction of gravity is obtained during the initialization of the visual inertial odometer, the gravity acceleration $g^{W}$ is not used as a variable to be optimized.

### 2.6. Wheeled Odometer Constraints

On ground mobile robots, a wheel speed meter is typically used to perform dead reckoning to obtain continuous relative poses of the robot. The continuous position and reliable scale estimation of the wheel odometer make it suitable for tasks such as path planning and navigation.

#### 2.6.1. Two-Dimensional Wheel Odometer Algorithm

The two-dimensional wheel odometer has an unavoidable cumulative, error but can provide a continuous carrier trajectory. As the wheel speed meter measures the average wheel speed over a period of time, the chassis speed measurement ${\hat{m}}_{base}$ measures the average speed during this time. The position and attitude update methods of the wheel speed odometer include Euler points, median points, and higher-order Runge–Kutta methods. As the sampling speed of the wheel speed meter is high (1 kHz), the Euler integration method is used to reduce the calculation time of the main control microcontroller. This is achieved while assuming that the chassis moves with fixed direction and speed in the original direction during the period and rotates to a new direction at the end of the time period.

The initial state of the wheel odometer is ${\hat{m}}_{odom0}={\left[p_{x0}\;p_{y0}\;\theta_{0}\right]}^{T}=0$. Given the previous state ${\hat{m}}_{odomk-1}={\left[p_{xk-1}\;p_{yk-1}\;\theta_{k-1}\right]}^{T}$ of the wheel odometer, the current chassis speed measurement ${\hat{v}}_{basek}={\left[v_{xk}\;v_{yk}\;\omega_{k}\right]}^{T}$, and the time difference $dt_{k}=t_{k}-t_{k-1}$, we can obtain the new wheel odometer state ${\hat{m}}_{odomk}$ as:

(11) $${\hat{m}}_{odomk}={\left[p_{xk}\;p_{yk}\;\theta_{k}\right]}^{T}$$

(12) $$\left[\begin{array}{c} p_{xk}\\ p_{yk} \end{array}\right]=\left[\begin{array}{c} p_{xk-1}\\ p_{yk-1} \end{array}\right]+\left[\begin{array}{cc} cos\theta_{k-1} & -sin\theta_{k-1}\\ sin\theta_{k-1} & cos\theta_{k-1} \end{array}\right]\left[\begin{array}{c} v_{xk}\\ v_{yk} \end{array}\right]dt_{k}$$

(13) $$\theta_{k}=\theta_{k-1}+\omega_{k}dt_{k}$$

#### 2.6.2. Wheel Odometer Pre-Integration

In this paper, we propose a pre-integration method for wheel odometer. We use IMU and wheel speed to measure the relative pose between two key frames. The data of the two sensors makes pre-fusion ${\hat{m}}_{fused\;odom}$. Thereafter, only the ${\hat{m}}_{fused\;odom}$ is measured, according to the wheel odometer kinematics equation, and a continuous calculation and integration is made to find the displacement.

The incremental update equation of the wheel odometer is:

(14) $$\{\begin{array}{c} p_{O_{i+1}}^{O_{k}}=p_{O_{i}}^{O_{k}}+R\left\{q_{O_{i}}^{O_{k}}\right\}\Delta p_{O_{i+1}}^{O_{i}}=p_{O_{i}}^{O_{k}}+R\left\{q_{O_{i}}^{O_{k}}\right\}\left(\Delta {\hat{p}}_{O_{i+1}}^{O_{i}}+D_{O_{k+1}}^{O_{k}}\eta_{os}\right)\\ q_{O_{i+1}}^{O_{k}}=q_{O_{i}}^{O_{k}}⊗q_{O_{i+1}}^{O_{i}}=q_{O_{i}}^{O_{k}}⊗q\left\{R_{B}^{O}\left({\hat{\omega }}_{avgi+1}-b_{g}-\eta_{g}\right)\Delta t\right\}\\ b_{gi+1}=b_{gi}+\eta_{b_{g}}\Delta t \end{array}$$

where $\Delta {\hat{p}}_{O_{i+1}}^{O_{i}}≜{\left[\Delta {\hat{p}}_{xi+1}\;\Delta {\hat{p}}_{yi+1}\;0\right]}^{T}$ denotes wheel odometer with noise measurement; the initial state value $p_{0}=0,\;q_{0}={\left[1\;0\;0\;0\right]}^{T}$. $x={\left[p^{O_{k}}\;q^{O_{k}}\;b_{g}\right]}^{T}$ is the pre-credit term.

The nominal weight of the wheel odometer pre-integration item can be incrementally updated based on the pre-fusion wheel odometer measurement:

(15) $$\{\begin{array}{c} {\hat{p}}_{O_{i+1}}^{O_{k}}={\hat{p}}_{O_{i}}^{O_{k}}+R\left\{{\hat{q}}_{O_{i}}^{O_{k}}\right\}\Delta {\hat{p}}_{O_{i+1}}^{O_{i}}\\ {\hat{q}}_{O_{i+1}}^{O_{k}}={\hat{q}}_{O_{i}}^{O_{k}}⊗{\hat{q}}_{O_{i+1}}^{O_{i}}={\hat{q}}_{O_{i}}^{O_{k}}⊗\hat{q}\left\{R_{B}^{O}\left({\hat{\omega }}_{avgi+1}-b_{g}\right)\Delta t\right\}\\ {\hat{b}}_{gi+1}={\hat{b}}_{gi} \end{array}$$

The initial value of nominal weight: ${\hat{p}}_{0}=0,\;{\hat{q}}_{0}={\left[1\;0\;0\;0\right]}^{T}$.

According to the definition of the error amount of the pre-integration term, an incremental update formula of the error amount of the pre-integration term is:

(16) {δpOi+1Ok=δpOiOk+R{qOiOk}DOk+1OkηosδθOi+1Ok=δθOiOk−(ω^avgi+1−bg)^δθOiOkΔt−δbgΔt+ηgΔt}δbgi+1=δbgi+ηbgΔt

(·)^ is 3×3 anti-symmetric matrix of Lie algebra SO (3). The nominal value of the wheel odometer pre-integration term depends on the pre-fusion wheel odometer measurement and the gyroscope zero offset. For the variable to be optimized, the zero bias of the gyroscope needs to be continuously adjusted in the pose measurement process to reduce the residual error. Therefore, in the optimization process, the partial derivatives $J_{b_{g}}^{p}$ and $J_{b_{g}}^{\theta }$ of the nominal value of the pre-integral term of the wheel odometer with respect to the zero offset of the gyroscope need to be used.

According to the incremental update of the error value of the pre-integration term of the wheel odometer, the Jacobian matrix of the error value between the two frames before and after can be obtained as:

(17) $$J_{\delta x_{i}}^{\delta x_{i+1}}=\frac{\delta x_{i+1}}{\delta x_{i}}=I+F_{i}$$

According to the definition of the nominal value of the pre-integration item of the wheel odometer, the Jacobian matrix of the error value is the nominal Jacobian matrix of $J_{{\hat{x}}_{i}}^{{\hat{x}}_{i+1}}=J_{\delta x_{i}}^{\delta x_{i+1}}$. Therefore, according to the chain-derivation rule, $J_{{\hat{x}}_{0}}^{{\hat{x}}_{i+1}}=J_{{\hat{x}}_{i}}^{{\hat{x}}_{i+1}}J_{{\hat{x}}_{0}}^{{\hat{x}}_{i}}$, the update equation of the nominal value of the pre-integration term of the wheel odometer with respect to the zero-biased Jacobian matrix is:

(18) $$J_{{\hat{x}}_{0}}^{{\hat{x}}_{i+1}}=\left(I+F_{i}\right)J_{{\hat{x}}_{0}}^{{\hat{x}}_{i}}$$

The initial value of the Jacobian matrix: $J_{0}=J_{{\hat{x}}_{0}}^{{\hat{x}}_{0}}=diag\left(9\right)$.

#### 2.6.3. Residual Term

Definition: In the least-squares problem of robot pose measurement, the wheel odometer residual term $r_{O}$ represents the error distance between the frame-to-frame relative displacement ${\hat{p}}_{O_{k+1}}^{O_{k}}$ and the key frame displacement $p_{O_{k+1}}^{O_{k}}$ in the variable to be optimized, where ${\hat{p}}_{O_{k+1}}^{O_{k}}$ is the observation, and $p_{O_{k+1}}^{O_{k}}$ is the estimator.

(19) $$r_{O}\left({\left\{\Delta {\hat{m}}_{odomt},{\hat{\omega }}_{avgt}\right\}}_{t_{k}\leq t\leq t_{k+1}},x_{k},x_{k+1}\right)=\left[\delta p_{O_{k+1}}^{O_{k}}\right]=p_{O_{k+1}}^{O_{k}}-{\hat{p}}_{O_{k+1}}^{O_{k}}$$

The wheel odometer residual does not include the errors $\delta \theta_{O_{k+1}}^{O_{k}}\;$ and $\delta b_{g}{}_{O_{k+1}}^{O_{k}}$ with respect to the rotation and gyro zero offset. This is as these terms are already defined in the residual term of the IMU pre-integration. The IMU pre-integration uses the original IMU measurement as the angular velocity input, which provides higher rotational integration accuracy than the wheel odometer pre-integration measured with a lower frequency pre-fused wheel odometer.

To use the variable *x*_*k*_, *x*_*k*+1_ to represent the wheel odometer pre-integration residual term, $p_{O_{k+1}}^{O_{k}}$ needs to be transformed:

(20) $$\begin{array}{cc} p_{O_{k+1}}^{O_{k}} & =R_{W}^{O_{k}}\left(p_{O_{k+1}}^{W}-p_{O_{k}}^{W}\right)\\ & =R_{O}^{B-1}R\left\{q_{B_{k}}^{W-1}\right\}\left(p_{B_{k+1}}^{W}-p_{B_{k}}^{W}\right)+R_{O}^{B-1}R\left\{q_{B_{k}}^{W-1}\right\}R\left\{q_{B_{k+1}}^{W}\right\}p_{O}^{B}-R_{O}^{B-1}p_{O}^{B} \end{array}$$

We obtain the residual term expressed using only the variables to be optimized and the wheel odometer pre-integration:

(21) $$r_{O}=R_{O}^{B-1}R\left\{q_{B_{k}}^{W-1}\right\}\left(p_{B_{k+1}}^{W}-p_{B_{k}}^{W}\right)+R_{O}^{B-1}R\left\{q_{B_{k}}^{W-1}\right\}R\left\{q_{B_{k+1}}^{W}\right\}p_{O}^{B}-R_{O}^{B-1}p_{O}^{B}-{\hat{p}}_{O_{k+1}}^{O_{k}}$$

Here, $R_{O}^{B}$ and $p_{O}^{B}$ are known constants.

As a maximum posterior problem, the robot pose measurement is the same as least-squares by introducing a covariance matrix of the residuals to transform the residuals with dimensions into a unified probability representation. The wheeled odometer residual $r_{O}$ obeys the covariance matrix $\Sigma_{O}$ of the wheeled odometer pre-integration, $r_{O}\sim N\left(0,\;{\left[\Sigma_{O}\right]}_{p}\right)$. Here, ${\left[\Sigma_{O}\right]}_{p}$ represents the displacement covariance in the wheel odometer pre-integration covariance matrix $\Sigma_{O}$, ${\left[\Sigma_{O}\right]}_{p}={\left[\Sigma_{O}\right]}_{left\;right\;3\times 3}$.

Regarding the Jacobian matrix, according to the definition of the wheel odometer residual $r_{O}$, in the optimization process, the residual value $r_{O}$ will change with the adjustment of the previous key frame poses $p_{B_{k}}^{W}$ and $q_{B_{k}}^{W}$, and the poses $p_{B_{k+1}}^{W}$ and $q_{B_{k+1}}^{W}$ of the next key frame, and the gyroscope zero offset $b_{gi}$ of the previous frame. To provide the necessary gradient direction for optimization, the system needs to be linearized in the current state $x_{k},x_{k+1}$ and the ratio between the increment of the residual $\partial r_{O}$ and the increment of the variable to be optimized is calculated. Thus, the Jacobian matrix $J_{x_{k},x_{k+1}}^{r_{O}}$ is defined:

(22) $$\partial r_{O}=J_{x_{k},x_{k+1}}^{r_{O}}\left[\begin{array}{c} \partial x_{k}\\ \partial x_{k+1} \end{array}\right]$$

(23) $$\left[\delta p_{O_{k+1}}^{O_{k}}\right]=J_{x_{k},x_{k+1}}^{r_{O}}\left[\begin{array}{c} \delta p_{B_{k}}^{W}\\ \delta v_{B_{k}}^{W}\\ \begin{array}{c} \delta \theta_{B_{k}}^{W}\\ \delta b_{aB_{k}}\\ \begin{array}{c} \delta b_{gB_{k}}\\ \delta p_{B_{k}}^{W}\\ \begin{array}{c} \delta v_{B_{k}}^{W}\\ \delta \theta_{B_{k}}^{W}\\ \begin{array}{c} \delta b_{aB_{k}}\\ \delta b_{gB_{k}} \end{array} \end{array} \end{array} \end{array} \end{array}\right]$$

Here, $J_{x_{k},x_{k+1}}^{r_{O}}\in R^{3\times 30}$. As the increment is small, using the quaternion definition will produce additional degrees of freedom. The increment for the rotation state in the formula is defined as the shaft angle representation.

As the wheel odometer residual is only related to some variables in the previous key frame state $x_{k}$ and next key frame state $x_{k+1}$, the value of the Jacobian matrix $J_{x_{k},x_{k+1}}^{r_{O}}$ is:

(24) $$J_{x_{k},x_{k+1}}^{r_{O}}=\left[\frac{\partial \delta p_{O_{k+1}}^{O_{k}}}{\partial \delta p_{B_{k}}^{W}}\;0\;\frac{\partial \delta p_{O_{k+1}}^{O_{k}}}{\partial \delta \theta_{B_{k}}^{W}}\;0\;\frac{\partial \delta p_{O_{k+1}}^{O_{k}}}{\partial \delta b_{gi}}\;\frac{\partial \delta p_{O_{k+1}}^{O_{k}}}{\partial \delta p_{B_{k+1}}^{W}}\;0\;\frac{\partial \delta p_{O_{k+1}}^{O_{k}}}{\partial \delta \theta_{B_{k+1}}^{W}}\;0\;0\right]$$

(25) ∂δpOk+1Ok∂δpBkW=−ROB−1R{qBkW−1}∂δpOk+1Ok∂δθBkW=−ROB−1[R{qBkW−1}(pBk+1W−pBkW+R{qBk+1W}pOB)]^∂δpOk+1Ok∂δbgi=−Jbgp∂δpOk+1Ok∂δpBk+1W=ROB−1R{qBkW−1}∂δpOk+1Ok∂δθBk+1W=−ROB−1R{qBkW−1}R{qBk+1W}(pOB)^

### 2.7. Marginalization and Prior Constraints

The state of the key frame and its related observations are constantly removed from the optimization equation. If all observations related to the removed key frames are directly discarded, the constraints of state estimation will be reduced, and the loss of valid information will lead to a decrease in accuracy. Here, a marginalization algorithm is used, while removing the key frames, retaining the removed observations to constrain the optimization variables. According to [6], the use of the Gauss–Newton method can be understood as adding an increment to the variable to be optimized; the objective function is the smallest.

If the residual function r(x) is linearized at x, and the Jacobian matrix $J_{x}^{r}$ is obtained, the nonlinear least-squares problem becomes a linear least-squares problem:

(26) $$\underset{\delta x}{\min}{\left\|r\left(x+\delta x\right)\right\|}^{2}⟹\underset{\delta x}{\min}{\left\|r\left(x\right)+J_{x}^{r}\delta x\right\|}^{2}\;$$

Here, ${\left\|r\left(x\right)+J_{x}^{r}\delta x\right\|}^{2}={\left[r\left(x\right)+J_{x}^{r}\delta x\right]}^{T}\left[r\left(x\right)+J_{x}^{r}\delta x\right]$. Taking the derivative of this formula with respect to δx be 0, we obtain:

(27) $$J_{x}^{r}{}^{T}J_{x}^{r}\delta x=-J_{x}^{r}{}^{T}r\left(x\right)$$

Let $H=J_{x}^{r}{}^{T}J_{x}^{r},\;\;b=-J_{x}^{r}{}^{T}r\left(x\right)$; thus, we obtain the incremental equation Hδx=b, where H is called the Hessian matrix.

Divide the variable x to be optimized into the part $x_{a}$, which needs to be removed, and the part $x_{b}$, $\delta x={\left[{\delta x}_{a}{\;\delta x}_{b}\right]}^{T}$, which needs to be retained, then the incremental equation becomes:

(28) $$\left[\begin{array}{cc} H_{a} & H_{b}\\ H_{b}{}^{T} & H_{c} \end{array}\right]\left[\begin{array}{c} \delta x_{a}\\ \delta x_{b} \end{array}\right]=\left[\begin{array}{c} b_{a}\\ b_{b} \end{array}\right]$$

The Schur method is used to eliminate the element to obtain the solution of $\delta x_{b}$:

(29) $$\left[\begin{array}{cc} I & 0\\ -H_{b}^{T}H_{a}^{-1} & I \end{array}\right]\left[\begin{array}{cc} H_{a} & H_{b}\\ H_{b}{}^{T} & H_{c} \end{array}\right]\left[\begin{array}{c} \delta x_{a}\\ \delta x_{b} \end{array}\right]=\left[\begin{array}{cc} I & 0\\ -H_{b}^{T}H_{a}^{-1} & I \end{array}\right]\left[\begin{array}{c} b_{a}\\ b_{b} \end{array}\right]$$

(30) $$\left[\begin{array}{cc} H_{a} & H_{b}\\ 0 & H_{c}-H_{b}^{T}H_{a}^{-1}H_{b} \end{array}\right]\left[\begin{array}{c} \delta x_{a}\\ \delta x_{b} \end{array}\right]=\left[\begin{array}{c} b_{a}\\ b_{b}-H_{b}^{T}H_{a}^{-1}b_{a} \end{array}\right]$$

Intercepting the second row of the above matrix, we obtain:

(31) $$\left(H_{c}-H_{b}^{T}H_{a}^{-1}H_{b}\right)\delta x_{b}=b_{b}-H_{b}^{T}H_{a}^{-1}b_{a}$$

In the above formula, only $x_{b}$ is unknown, and no information in H and b is lost. This process removes the rows and columns related to $x_{a}$ from the incremental equation, marginalizes the state $x_{a}$ that needs to be removed, and retains the historical observation constraints on the state $x_{b}$. When the next image frame arrives, the prior information in the above Formula (31) will be used as a prior constraint term to construct a nonlinear least-squares problem.

### 2.8. State Initialization

VINS-Mono uses multiple steps to initialize the state: gyroscope zero offset correction, initializing gravity, speed, and scale coefficients, and modifying the direction of gravity. The disadvantage of this method is that it depends on sufficient visual measurement of parallax and sufficient acceleration excitation. When there is no abnormal situation, such as skidding, the wheel odometer has better accuracy and reliability over a short distance and a short duration. Compared with monocular vision, there is no scale uncertainty, and it is easier to initialize the keyframe pose, velocity, and gravity directions.

#### 2.8.1. Gyro Zero Offset Initialization

As the gyroscope and wheel odometer measurements are on the same rigid body, the rotations of the two are the same. The relative rotation between the two key frames can be obtained through IMU pre-integration and wheel odometer pre-integration, respectively: $q_{B_{k+1}}^{B_{k}}$ and $q_{O_{k+1}}^{O_{k}}$. The rotation term of the pre-integration of the wheeled odometer above is also obtained through the gyro integration and has no reference value. Therefore, during the initialization process, the gyroscope pre-integration will use the heading angle for rotation integration. Rotation term $q_{B_{k+1}}^{B_{k}}$ is a function of the gyroscope bias $q_{B_{k+1}}^{B_{k}}$. If the error between $q_{B_{k+1}}^{B_{k}}$ and $q_{O_{k+1}}^{O_{k}}$ is used as a constraint, the gyroscope bias $b_{g}$ can be estimated. Assuming that the gyro bias $b_{gk}$ of each key frame during the initialization process is the same $b_{gk}=b_{g}$, the construction of the least-squares problem is as follows:

(32) $$b_{g}{}^{*}=\underset{b_{g}}{\mathrm{argmin}}\overset{k-1}{\underset{i=1}{\sum }}{\left\|q_{O}^{B}⊗{\hat{q}}_{O_{i+1}}^{O_{i}}{}^{-1}⊗q_{B}^{O}⊗q_{B_{k+1}}^{B_{k}}\right\|}^{2}$$

Linearizing the rotation transform at ${\hat{q}}_{B_{k+1}}^{B_{k}}$, we obtain:

(33) $$q_{B_{k+1}}^{B_{k}}={\hat{q}}_{B_{k+1}}^{B_{k}}⊗q\left\{J_{b_{g}}^{q}b_{g}\right\}\approx {\hat{q}}_{B_{k+1}}^{B_{k}}⊗\left[\begin{array}{c} 1\\ \frac{1}{2}J_{b_{g}}^{q}b_{g} \end{array}\right]$$

Here, $J_{b_{g}}^{q}$ is the partial derivative of the inter-frame rotation $q_{B_{k+1}}^{B_{k}}$. $b_{g}$ is the gyroscope zero bias. The objective function of the least-squares problem is written as:

(34) $$\begin{array}{cc} q_{O}^{B}⊗{\hat{q}}_{O_{i+1}}^{O_{i}}{}^{-1}⊗q_{B}^{O}⊗q_{B_{k+1}}^{B_{k}} & =\left[\begin{array}{c} 1\\ 0 \end{array}\right]\\ {\hat{q}}_{B_{k+1}}^{B_{k}}⊗\left[\begin{array}{c} 1\\ \frac{1}{2}J_{b_{g}}^{q}b_{g} \end{array}\right] & =q_{O}^{B}⊗{\hat{q}}_{O_{i+1}}^{O_{i}}⊗q_{B}^{O}\\ \left[\begin{array}{c} 1\\ \frac{1}{2}J_{b_{g}}^{q}b_{g} \end{array}\right] & ={\hat{q}}_{B_{k+1}}^{B_{k}}{}^{-1}⊗q_{O}^{B}⊗{\hat{q}}_{O_{i+1}}^{O_{i}}⊗q_{B}^{O} \end{array}$$

Considering only the imaginary part of the quaternion, we obtain:

(35) $$J_{b_{g}}^{q}b_{g}=2{\left\{{\hat{q}}_{B_{k+1}}^{B_{k}}{}^{-1}⊗q_{O}^{B}⊗{\hat{q}}_{O_{i+1}}^{O_{i}}⊗q_{B}^{O}\right\}}_{xyz}\;$$

The above Formula (35) conforms to the format of Hx=b, and the Cholesky decomposition can be used to find the least-squares solution:

(36) $$J_{b_{g}}^{q}{}^{T}J_{b_{g}}^{q}b_{g}=2J_{b_{g}}^{q}{}^{T}{\left\{{\hat{q}}_{B_{k+1}}^{B_{k}}{}^{-1}⊗q_{O}^{B}⊗{\hat{q}}_{O_{i+1}}^{O_{i}}⊗q_{B}^{O}\right\}}_{xyz}\;$$

If the robot rotates rapidly during the gyro work offset initialization process, the elasticity of the wheel, the rigid connection between the wheel and the IMU, the misalignment of the wheel odometer clock and the IMU clock, and a calibration error of the wheel odometer rotation scale factor may lead to poor gyro work offset initialization results.

#### 2.8.2. Initialization of Key Frame Speed and Gravity

As the Mecanum wheel will tremble during movement, and the wheeled odometer algorithm can only obtain the heading angle information, it is difficult to obtain accurate relative rotation between key frames through wheeled odometer integration. In the previous step, the zero offset of the gyroscope has been initialized, and the relative rotation between all key frames can be obtained through IMU pre-integration. As the rotation is known, the key frame speed and gravity can be calculated by solving linear equations. Decomposing the position term $\alpha_{B_{k+1}}^{B_{k}}$ and speed term $\beta_{B_{k+1}}^{B_{k}}$ in the IMU pre-integration and transforming it into the form of matrix multiplication ${\hat{z}}_{B_{k+1}}^{B_{k}}=H_{B_{k+1}}^{B_{k}}x_{B_{k+1}}^{B_{k}}$, we obtain:

(37) $$\left[\begin{array}{c} \alpha_{B_{k+1}}^{B_{k}}-p_{O}^{B}+R_{B_{k+1}}^{B_{k}}p_{O}^{B}\\ \beta_{B_{k+1}}^{B_{k}} \end{array}\right]=\left[\begin{array}{c} \begin{array}{ccc} -I\Delta t & 0 & \begin{array}{cc} \frac{1}{2}R_{B_{0}}^{B_{k}}\Delta t^{2} & R_{O}^{B}{\hat{p}}_{O_{k+1}}^{O_{k}} \end{array} \end{array}\\ \begin{array}{ccc} -I & R_{B_{k+1}}^{B_{k}} & \begin{array}{cc} R_{B_{0}}^{B_{k}}\Delta t & \;0 \end{array} \end{array}\; \end{array}\right]\left[\begin{array}{c} v_{B_{k}}^{B_{k}}\\ \begin{array}{c} v_{B_{k+1}}^{B_{k+1}}\\ g^{B_{0}}\\ s \end{array} \end{array}\right]$$

Here, ${\hat{z}}_{B_{k+1}}^{B_{k}}$ is the IMU pre-integration measurement between the key frames $B_{k}\;$ and $B_{k+1}$. The variable to be estimated related to the key frames $B_{k}\;$ and $B_{k+1}$ is defined as $x_{B_{k+1}}^{B_{k}}$, and $H_{B_{k+1}}^{B_{k}}\;$ represents the constraint between the measurement ${\hat{z}}_{B_{k+1}}^{B_{k}}\;$ and the $x_{B_{k+1}}^{B_{k}}$. The s in the $x_{B_{k+1}}^{B_{k}}$ represents the distance of the wheel odometer with respect to the actual distance, that is, the X-axis and Y-axis scale factors of the wheel odometer $S_{x},S_{y}$. If the IMU excitation is sufficient, it can be used to calibrate the scale factor. S=1 is defined here for the reliability of initialization.

To reduce initialization errors and improve reliability, multiple key frame measurements need to be used as constraints to calculate the key frame speed and gravity direction. By combining the multiple linear equations above, we can obtain the least-squares problem:

(38) $$x^{*}=\underset{x}{\mathrm{argmin}}\underset{k\in Frames}{\sum }{\left\|{\hat{z}}_{B_{k+1}}^{B_{k}}-H_{B_{k+1}}^{B_{k}}x_{B_{k+1}}^{B_{k}}\right\|}^{2},x=\left[\begin{array}{c} v_{B_{0}}^{B_{0}}\\ v_{B_{1}}^{B_{1}}\\ \begin{array}{c} \vdots \\ v_{B_{k}}^{B_{k}}\\ \begin{array}{c} g^{B_{0}}\\ s \end{array} \end{array} \end{array}\right]$$

Here, *x* is the variable to be estimated, and $x^{*}$ is the optimal estimated value of *x*. The program uses Cholesky decomposition to solve the least-squares problem:

(39) $$H^{T}Hx=H^{T}\hat{z}$$

In the formula, the matrix H is obtained by inserting all $H_{B_{k+1}}^{B_{k}}\;$ into empty columns at the corresponding positions of the unrelated variables and summing them, and $\hat{z}$ is obtained by combining all ${\hat{z}}_{B_{k+1}}^{B_{k}}$.

## 3. Experiments

In this paper, a mobile robot platform is built, using a dual-processor architecture. The high-performance PC upper computer is used for data processing of each sensor and the SLAM algorithm operation, and the embedded controller lower computer is used for the motion control of the mobile robot chassis. The parameters of the high-performance PC are Intel Core i7-9750H CPU @ 2.6 GHz, 16 GB RAM. The camera is IntelRealSense ZR300.

### 3.1. Accuracy Verification Experiment

#### 3.1.1. Room-Scale Pose Measurement Experiment

**Figure 2:**
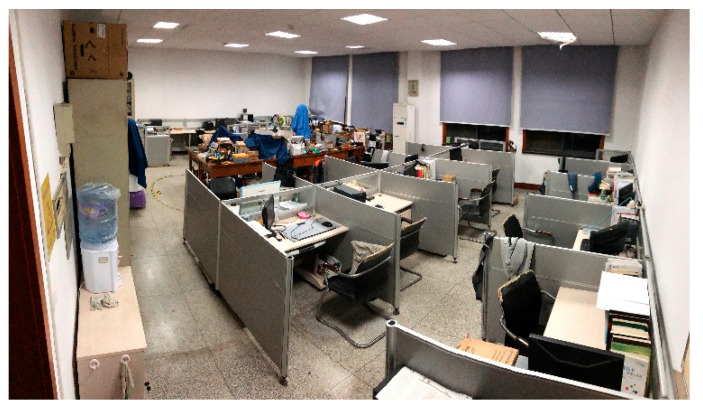
Room-scale experimental environment.

Experimental conditions: In a laboratory where objects are placed in a complex environment, as shown in Figure 2, the control robot walked through all the channels. The channel width is narrow, and the width at the narrowest point is less than 1 m; the movement speed was maintained at approximately 0.5 m/s; a large number of fixed marking points are arranged inside the room to obtain the real position of the robot in order to analyze the positioning error of the robot. Abnormal conditions during the experiment included:

3. magnetic guide bars with a height of approximately 0.5 cm were fixed on the ground, and the wheels slipped slightly as they passed;
4. due to turning too close to the weakly textured wall surface, the visual tracking was completely lost several times.

**Figure 3:**
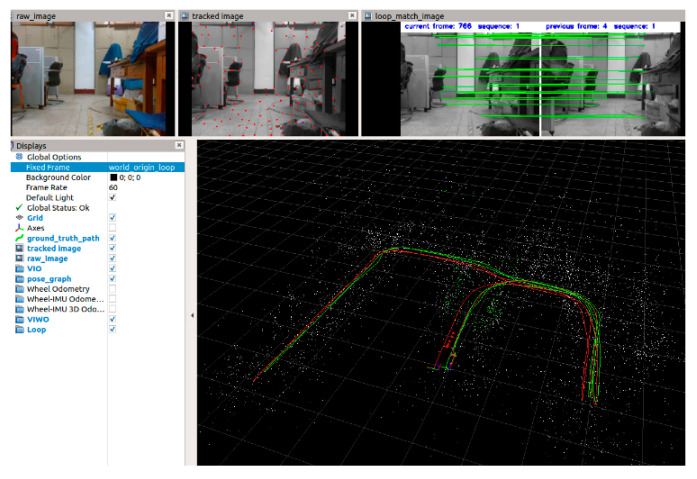
RViz interface at the end of test data playback.

The RViz interface at the end of the test data playback is shown in Figure 3, which includes the raw picture, feature points, matched feature points, and the path of pose measurement.

**Figure 4:**
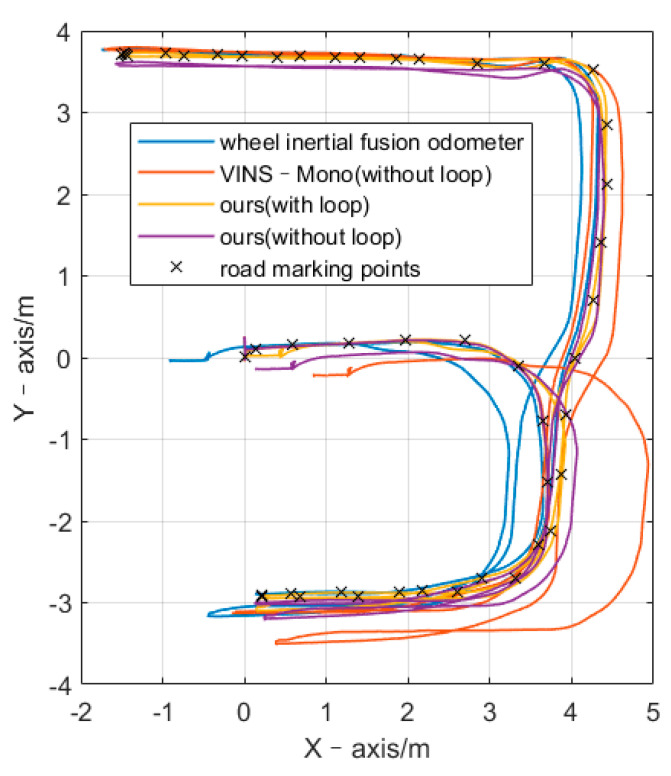
Path of pose measurement at room scale.

The path diagrams of pose measurement, as shown in Figure 4, start along the positive X-axis. Therefore, the distance between the start point and end point shows the experimental accuracy. Throughout the experiment, the car traveled for 184.3 s, the average speed was 0.264 m/s, the maximum speed was 0.591 m/s, the cumulative translation was 51.321 m, the cumulative rotation was 3428.318°, and the chassis had no abnormalities. As this experiment was performed in an actual scene, the experimental system did not have the equipment to measure ground truth; therefore, it could only measure the distance error of the car passing several fixed road marking points. In each experiment, the robot was controlled to accurately pass the road marking point and keep the rotation direction consistent. Therefore, the pose error in Table 1 is average position error and yaw angle error among several road marking points. When the robot passes through each road marking point, a timestamp is marked. Calculate the coordinate error (∆X and ∆Y), position error (∆P(m), absolute value), and yaw angle error (∆A) between the algorithm trajectory pose and the road marking point when it is at the time stamp. For each algorithm, take the average of the errors generated by all road marking points as the final experimental result. The position error rate (∆P(%)) is the ratio of the position error to the cumulative translation. The table shows our algorithm performed best, with and without closed loops. The position error was 0.206 m and 0.015 m, respectively. As shown in Figure 4, we can clearly see the error of the different methods.

As listed in Table 1, we compared the error of the X-axis, Y-axis, position, and heading angle. According to the data presented in Table 1, the accuracy of posture estimation using the monocular vision inertial wheel odometer algorithm was better than that of the VINS-Mono algorithm, and the accumulated position error was only approximately 0.2 m after 51 m. We divided the cumulative position error by the cumulative translation distance to obtain the cumulative position error rate. The position error rate of the proposed algorithm was only 0.4%, which was lower than that of VINS-Mono (1%). This experiment verified that a multi-sensor fusion odometer algorithm could perform high-precision positioning in a room-scale indoor environment, and the result was better than that of the monocular and wheel separately. By further combining complete SLAM system, ours almost have no bias.

#### 3.1.2. Floor Scale Experiment

Experimental conditions included:

① On the first floor of the building where the laboratory was located, the floor area was approximately 250 × 100 m;
② The robot ran in the central hall with 1 m/s (the area of the hall was approximately 15 × 15 m);
③ A large number of fixed marking points are arranged inside the corridor to obtain the real position of the robot in order to analyze the positioning error of the robot.

Anomalies during the experiment included:

① There was a considerable amount of dust on the ground, which decreased the friction between the wheels, leading to a slight slip during rapid turns and a significant slip during left-to-right translation;
② Due to the fast movement of the robot, the picture of the rolling shutter camera continued to exhibit disturbances;
③ There were cable manhole covers in numerous places in the corridor, the ground surface was uneven, and there were 2–3 cm step-like undulations. The robot vibrated significantly while passing over these obstacles;
④ The corridor contained semi-open areas and closed areas, the environment brightness changed significantly, and some areas were almost completely dark;
⑤ The walls around the robot in some areas were covered with tiles and had reflections;
⑥ The corridor and hall scenes had a high degree of similarity, lacking special landmarks. Loop detection was successfully performed only when the robot passed the hall halfway and finally returned to the hall;
⑦ During the experiment, pedestrians appeared several times.

**Figure 5:**
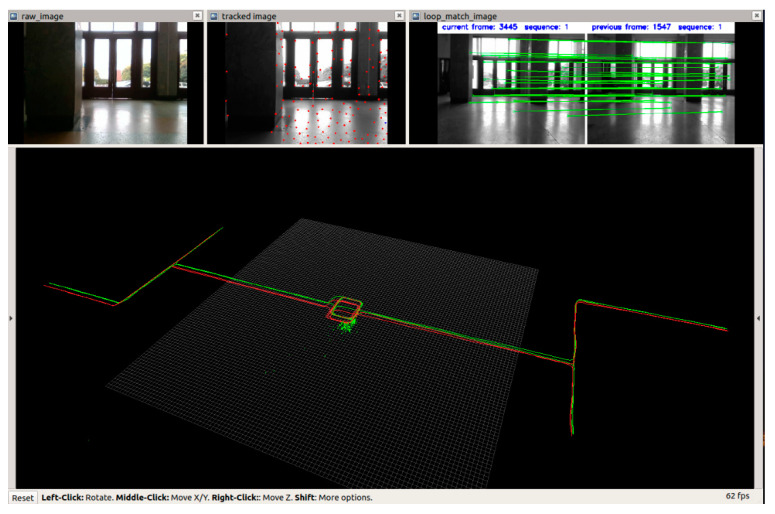
RViz interface at the end of test data playback.

The RViz interface at the end of test data playback is shown in Figure 5, which includes the raw picture, feature points, matched feature points, and the path.

**Figure 6:**
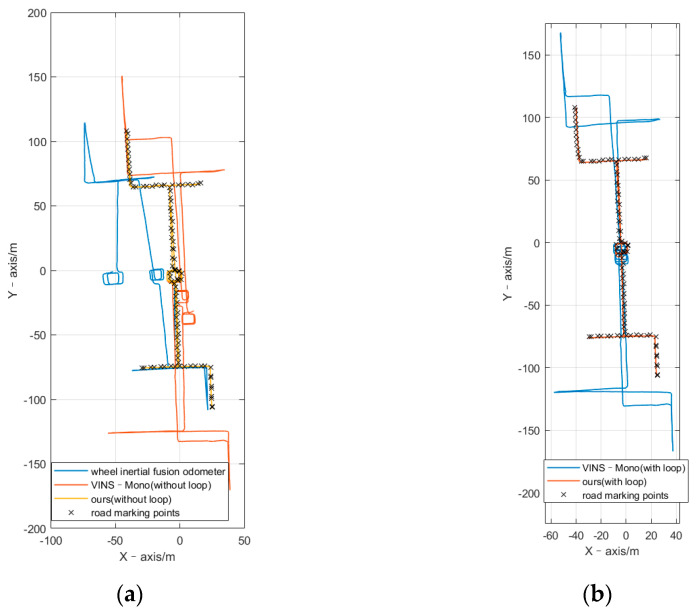
(**a**) Path of floor-scale without loop and (**b**) path of floor-scale with loop.

Throughout the experiment, the car traveled for 889.2 s, the average speed was 0.87 m/s, the maximum speed was 1.36 m/s, the cumulative translation was 809.27 m, the cumulative rotation was 15,502.1°, and the chassis abnormal time was 26.983 s. As shown in Figure 6 and Table 2, the uneven ground made the wheel slip, so the error was very high. As shown in the floor-scale scenario, both the VINS-Mono algorithm with loop detection disabled and ours performed better; however, there was still a cumulative positioning error that could not be neglected. Compared with the complete SLAM system, VINS-Mono and ours both significantly improved the accuracy. Additionally, ours was better than that of VINS-Mono.

### 3.2. Robustness Verification Experiment

This time, we created experiments with sensor measurement errors or even loss. Experimental conditions included intentionally blocking the camera, resulting in the visual signal being lost for approximately 15 s. This was undertaken in the laboratory, during the movement of the robot, during which the robot kept moving and turning.

**Figure 7:**
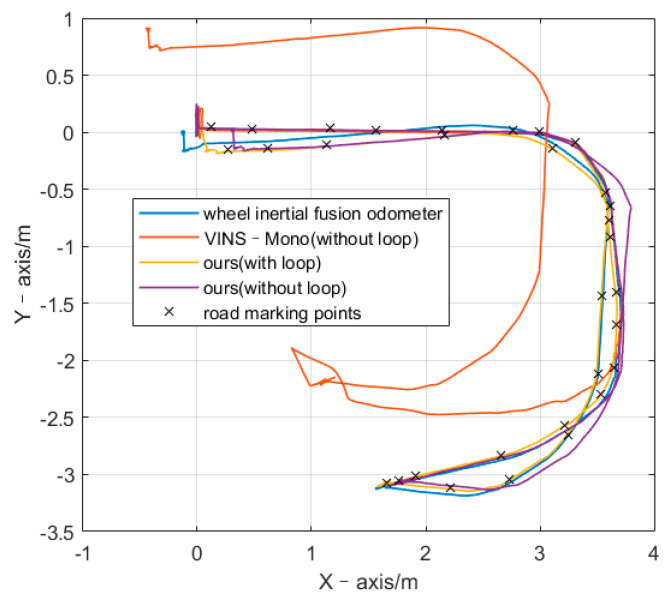
Path while visual tracking is failed.

Throughout the experiment, the car traveled for 104.4 s, the average speed was 0.142 m/s, the maximum speed was 0.803 m/s, the cumulative translation was 17.809 m, the cumulative rotation was 1110.32°, and the chassis had no abnormalities. In the process of visual measurement loss, upon which the VINS-Mono is based, the state estimator was downgraded to inertial navigation dead reckoning. The error increased rapidly, thereby reducing the final positioning accuracy. As shown in Figure 7 and Table 3 during the loss of visual features, the path of the SLAM algorithm was the same as that measured by the wheel odometer. This shows that, although the positioning accuracy was affected by the loss of visual measurement, the pre-integration constraint can still provide absolute speed measurement; thus, the final positioning error was less than that of VINS-Mono.

## 4. Discussion

Aiming to solve the problems of low accuracy and poor robustness of the visual inertial SLAM algorithm, we designed and implemented a tightly coupled monocular visual inertial pose estimation algorithm that integrates wheel speed information. The state estimator, based on tightly coupled multi-sensor information, is used as the core of the algorithm, and the wheel odometer pre-integration that integrates wheel speed and IMU angular velocity avoids repeated integration in the iterative optimization process. The pose of the robot in the sliding window and the visual feature points are the state to be estimated. Then, the state is initialized based on the wheel odometer and IMU, so that the initial value of the state can be calculated quickly and reliably in both stationary and moving states. Finally, the residual constraint is added to the state estimator, and the optimal robot pose is solved through nonlinear optimization. Compared with the existing SLAM algorithm for many experiments, the SLAM algorithm in this paper can achieve higher pose accuracy and robustness in environments with more extreme situations. Experimental comparison results show that the SLAM algorithm proposed in this paper is feasible and effective.

In future research, our algorithm can be improved in the following ways. First, a motion capture system or D-GPS can be employed to obtain ground truth for quantitative pose comparison analysis. Second multiple monocular cameras with non-common view relationships can be used, which can improve robustness. When some cameras are blocked, the other cameras can still provide reliable visual measurement. Further, based on the robust robot pose measurement, using binocular or depth cameras to build a dense map of the environment can meet the requirements of robot navigation and obstacle avoidance.

## Figures and Tables

**Table 1:** Room-scale pose measurement results.

Algorithm	∆X (m)	∆Y (m)	∆P (m)	∆P (%)	∆A (°)
wheel	−0.785	−0.389	0.87	1.71	1.907
wheel inertial	−0.905	−0.040	0.906	1.77	−0.507
VINS-Mono	0.851	−0.220	0.879	1.71	−0.574
VINS-Mono (loop)	0.009	0.020	0.022	0.04	−0.530
ours	0.148	−0.143	0.206	0.40	−0.213
ours (loop)	−0.003	0.015	0.015	0.03	−0.443

**Table 2:** Floor-scale pose measurements results.

Algorithm	∆X (m)	∆Y (m)	∆P (m)	∆P (%)	∆A (°)
wheel	−83.768	0.295	83.768	10.31	32.868
wheel inertial	−52.108	−0.826	52.115	6.42	6.404
VINS-Mono	10.692	−31.422	33.191	4.09	4.080
VINS-Mono (loop)	0.138	−5.281	5.281	0.65	3.086
ours	−2.025	0.947	2.235	0.28	3.661
ours (loop)	−0.295	0.165	0.338	0.04	3.172

**Table 3:** Pose measurement results when visual tracking was lost.

Algorithm	∆X (m)	∆Y (m)	∆P (m)	∆P (%)	∆A (°)
wheel	−0.042	0.106	0.114	0.64	−1.078
wheel inertial	−0.137	−0.014	0.137	0.77	−2.811
VINS-Mono	−0.442	0.890	0.994	5.58	−2.057
VINS-Mono (loop)	−0.030	0.015	0.025	0.19	−1.599
ours	0.295	0.011	0.295	1.66	−2.883
ours (loop)	0.020	−0.014	0.023	0.14	−1.718

## Data Availability

The data presented in this study are available on request from the corresponding author. The data are not publicly available due to the data may involve confidential information of our research institution.

## Abbreviations

**Symbols**	**Meaning**
X	variables to be estimated
i,j,k	key frame
K	set of key frames
l	feature point
ℒ	set of feature points
$x_{k}$	state under key frame k
$\lambda_{l}$	inverse depth of feature point l
$p_{B}^{W}$	position(displacement coordinate of B relative to W)
$q_{B}^{W}$	attitude(quaternion form)
$v_{B}^{W}$	speed
$b_{a}$	accelerometer bias
$b_{g}$	gyroscope bias
${\hat{m}}_{odomk}$	wheel odometer under key frame k
$\hat{m}$	means term m is the observation
≜	defined as
$\text{\{}p_{xk}$$p_{yk}$}	coordinate of the wheel odometer under key frame k
$\theta_{k}$	angle of rotation of the wheel odometer under key frame k
Z	observation
$Z_{C_{i}}$	visual feature point observation under key frame i
${\hat{z}}_{il}$	feature points observed under key frame i
${ℬ}_{ij}$	IMU pre-integration between key frame i and key frame j
t	time
${\hat{a}}_{t}$	pre-integration of accelerated speed at time t
${\hat{\omega }}_{t}$	pre-integration of angular speed at time t
$O_{ij}$	pre-fusion wheel odometer between key frame i and key frame j
$X^{*}$	maximum posterior estimation of variables
p(Z|X)	conditional probability of observing the occurrence of Z under the existed state X
p(X)	prior probability (edge probability)
$\|r{\left(\cdot \right)\|}_{\Sigma }^{2}$	Mahalanobis distance
Σ	covariance matrix
$\left\{r_{p},H_{p}\right\}$	prior information from marginalization
$r_{C}$	visual residual
$r_{O}$	wheel odometer residual
$r_{ℬ}$	IMU residual
ρ(·)	Huber loss function
$\left(p_{C_{i}},q_{C_{i}}\right)$	spatial pose under key frame i
$r_{C_{jl}}$	error of the feature point l in the position of key frame j
$R_{{ℱ}_{2}}^{{ℱ}_{1}}$	rotation matrix from ${ℱ}_{2}$ coordinate system to ${ℱ}_{1}$ coordinate system
${{\hat{P}}_{l}}^{c_{j}}$	position where the feature point l is projected onto the unit ball in the key frame j
$\pi_{c}^{-1}$	back projection function
$C_{j}$	camera coordinate system of the key frame j
${{\hat{P}}_{l}}^{C_{j}}$	position projected on the unit ball in the key frame j
$\left[\begin{array}{cc} b_{1} & b_{2} \end{array}\right]$	two orthogonal base vectors on the tangent plane of the unit ball and the projection lines with the feature points in the orthogonal direction
${\hat{\alpha }}_{B_{k}+1}^{B_{k}},{\hat{\beta }}_{B_{k}+1}^{B_{k}},\;{\hat{q}}_{B_{k}+1}^{B_{k}}$	pre-integrated IMU measurement terms, observation α is position term; β is velocity term; q is rotation term
δ	represents the error term
$\delta \theta_{B_{k}+1}^{B_{k}}$	three-dimensional small perturbation
$p_{O_{i}}^{O_{k}},{\hat{p}}_{O_{i}}^{O_{k}}$	displacement between key frame k and key frame i(the term with “^” is the observation, without “^” is the estimate)
$\eta_{os}$	position measurement noise, Gaussian
$D_{O_{k+1}}^{O_{k}}$	noise variance coefficient between key frame k and key frame k+1
$\eta_{g}$	angular velocity white noise
$\eta_{b_{g}}$	angular velocity zero error
(·)^	3×3 anti-symmetric matrix of Lie algebra
$J_{\delta x_{i}}^{\delta x_{i+1}}$	Jacobian matrix of the error value between the two frames before and after
$J_{{\hat{x}}_{i}}^{{\hat{x}}_{i+1}}$	Jacobian matrix of the nominal
$R_{O}^{B}$	known constant
${\hat{z}}_{B_{k+1}}^{B_{k}}$	IMU pre-integration measurement between the key frames $B_{k}$ and $B_{k+1}$
$x_{B_{k+1}}^{B_{k}}$	variable to be estimated related to the key frames $B_{k}$ and $B_{k+1}$
$H_{B_{k+1}}^{B_{k}}$	constraint between the measurement ${\hat{z}}_{B_{k+1}}^{B_{k}}$ and the $x_{B_{k+1}}^{B_{k}}$
$S_{x},S_{y}$	X-axis and Y-axis scale factors of the wheel odometer
